# Deconvolution of the vestibular evoked myogenic potential using the power spectrum of the electromyogram

**DOI:** 10.1186/s12976-015-0018-x

**Published:** 2015-10-06

**Authors:** Bernd Lütkenhöner

**Affiliations:** ENT Clinic, Münster University Hospital, Kardinal-von-Galen-Ring 10, Münster, Germany

**Keywords:** EMG, MUAP, VEMP

## Abstract

**Background:**

The vestibular evoked myogenic potential (VEMP) can be modelled reasonably well by convolving two functions: one representing an average motor unit action potential (MUAP), the other representing the temporal modulation of the MUAP rate (rate modulation). It is the latter which contains the information of interest, and so it would be desirable to be able to estimate this function from a combination of the VEMP with some other data. As the VEMP is simply a stimulus-triggered average of the electromyogram (EMG), a supplementary, easily accessible source of information is the EMG power spectrum, which can be shown to be roughly proportional to the squared modulus of the Fourier transform of the MUAP. But no phase information is available for the MUAP so that a straightforward deconvolution is not possible.

**Methods:**

To get around the problem of incomplete information, the rate modulation is described by a thoughtfully chosen function with just a few adjustable parameters. The convolution model is then used to make predictions as to the energy spectral density of the VEMP, and the parameters are optimized using a cost function that quantifies the difference between model prediction and data.

**Results:**

The workability of the proposed approach is demonstrated by analysing Monte Carlo simulated data and exemplary data from patients who underwent VEMP testing as part of a clinical evaluation of their dizziness symptoms.

**Conclusions:**

The approach is suited, for example, to estimate the duration of the inhibition causing the VEMP or to disentangle a VEMP consisting of more than one component.

## Introduction

The otolith organs in the inner ear, saccule and utricle, are sensors for linear accelerations and, as such, are important for controlling posture and eye movement. To assess their function in clinical settings it can be exploited that they also respond to brief high-intensity sounds. Muscle reflexes elicited that way give rise to short-latency myogenic responses, which can be recorded using surface electrodes placed over the respective muscles. This type of signal is called vestibular evoked myogenic potential (VEMP). Two varieties of VEMP are to be distinguished: The cVEMP represents a vestibulo-collic reflex and is recorded from a cervical muscle such as the sternocleidomastoid, whereas the oVEMP represents a vestibulo-ocular reflex and arises from the extraocular muscles [1, 2]. The two signals provide complementary diagnostic information: While the sound-evoked cVEMP is assumed to originate primarily in the ipsilateral saccule, the oVEMP is attributed mainly to the contralateral utricle [3]. Only the cVEMP is considered in this article, but the theoretical framework developed is applicable to the oVEMP as well.

In clinical investigations, the data evaluation is normally confined to determining the amplitudes and latencies of the major peaks of the VEMP, which typically has a sterotyped waveform. However, a deeper understanding of the response can only be obtained with the help of a model. Wit and Kingma [4] modelled the electromyogram (EMG) as the sum of motor unit action potentials (MUAP) and explained the VEMP by a brief inhibitory modulation of the firing rates of the motor units. Building on this pioneering work, a convolution equation of the form

(1) $$  v(t) = c_{v} \int_{-\infty}^{\infty} r(t') h(t-t') dt',  $$

was derived, where *v*(*t*) is the VEMP, *h*(*t*) is the MUAP, *r*(*t*) is a function called the rate modulation, and *c*_*v*_ is a constant factor [5]. In the frequency domain this equation reads

(2) $$  V(f) = c_{v} R(f) H(f),  $$

where *V*, *R*, and *H* are the Fourier transforms of *v*, *r*, and *h*, respectively. The quantity of interest is *r*(*t*), and therefore the question arises as to how to estimate this quantity from the data. A method presented in a previous article [6] exploited the fact that *r*(*t*) effects not only the mean of the EMG, i.e., the VEMP, but causes also a modulation of the variance. Unfortunately, this VEMP-associated variance modulation is not generally workable in standard VEMP investigations, because it typically has a much lower signal to noise ratio than the VEMP itself (as yet, the method was applied only to grand-averaged data). In the present article we therefore exploit a different source of information which promises to be much more robust: the power spectrum of the EMG. A simulation conducted by Wit and Kingma [4] suggested that the overall shape of this spectrum corresponds to the energy density spectrum of the MUAP. Thus, by implication, the power spectrum of the electromyogram allows us to estimate |*H*(*f*)|, except for a factor that is essentially independent of frequency. Moreover, from Eq. (2) it follows that dividing |*V*(*f*)| by this estimate provides an estimate of |*R*(*f*)|, at least conceptually.

The problem remains that this procedure provides no phase information. A conceivable solution is to resort to higher-order spectra [7]. However, caution is advised because such spectra are highly susceptible to noise [8]. One might also consider reconstructing *H*(*f*) from its modulus by making certain assumptions about the phase. It could be assumed, for example, that *H*(*f*) represents, in good approximation, a minimum phase filter. However, such assumptions would not be well-founded. Therefore yet another approach is proposed here: Assumptions are made about *R*(*f*) rather than *H*(*f*), which appears to be a much easier task. It will be shown that this approach allows us, indeed, to interpret the VEMP using information from the EMG. Depending on the quality of the data, it becomes possible, for example, to estimate the duration of an inhibitory or excitatory modulation and to get hints as to a possible multi-component structure of the VEMP.

## Theory

### Power spectral density of the electromyogram

The relationship between VEMP and EMG spectrum was already addressed in the pioneering modeling study by Wit and Kingma [4]. Making the assumption that the MUAP waveform is the same for all motor units, the EMG was modelled as

(3) $$  s(t) = \sum_{n=1}^{N} a_{n} h(t-t_{n}),  $$

where *N* is the number of contributing MUAPs, and *t*_*n*_ and *a*_*n*_ specify the occurrence time and the amplitude, respectively, of the *n*-th MUAP (1≤*n*≤*N*). A time shift by *t*_*n*_ corresponds to a frequency-domain multiplication by $e^{-\mathfrak {i} 2\pi {f}{t_{n}}}$ [9]. Thus, the Fourier transform of *s*(*t*) is

(4) $$ S(f) = A_{N}(f) H(f)  $$

with

(5) $$  A_{N}(f) = \sum_{n=1}^{N} a_{n} e^{-\mathfrak{i} 2\pi{f}{t_{n}}}.  $$

A simulation by Wit and Kingma [4] suggests that |*S*(*f*)| gets its overall shape from the factor |*H*(*f*)| and its noisy character from the factor |*A*_*N*_(*f*)|.

Building on these ideas we will now derive a simple analytical formula for the expected power spectral density of the EMG. We first calculate the expectation of

(6) $$ |A_{N}(f)|^{2} = \left(\sum_{n=1}^{N} a_{n} e^{-\mathfrak{i} 2\pi{f}{t_{n}}} \right) \cdot \left(\sum_{n=1}^{N} a_{n} e^{\mathfrak{i} 2\pi{f}{t_{n}}} \right).  $$

By expanding the product on the right-hand side, gathering terms, and exploiting the relationship between exponential and cosine function, the equation can be rewritten as

(7) $$  |A_{N}(f)|^{2} = \sum_{n=1}^{N} {a_{n}^{2}} + 2 \sum_{n=1}^{N-1} \sum_{m=n+1}^{N} a_{n} a_{m} \cos\Big(2\pi f (t_{n}-t_{m})\Big).  $$

For the next step it is assumed that both the occurence times and the amplitudes of the MUAPs are identically distributed random variables, that all random variables are independent, and that the numbering of the MUAPs does not imply a specific order. These assumptions ensure that corresponding summands in Eq. (7) have the same expectation. The expectation of |*A*_*N*_(*f*)|^2^ can therefore be calculated from the expectations of representative summands. This way we get

(8) $$  E\left[\!|A_{N}(f)|^{2}\right] = N E[{a_{1}^{2}}] + N (N-1) E[a_{1}] E[a_{2}] E[\cos\big(2\pi f (t_{1}-t_{2})\big)].  $$

As to the cosine term we assume that the MUAP occurrence times are uniformly distributed between −*T*/2 and *T*/2, which means that the time difference between two distinct occurrence times (*t*_1_−*t*_2_ in Eq. (8)) has the probability density function

(9) $$ f_{\Delta{t}}(x) = \frac{1}{T} \left(1-\frac{|x|}{T}\right),  $$

|*x*|≤*T*. Calculation of the expectation of the cosine term and introducing the notations $\bar {a}$ and ${\bar {\bar {{a}}}}^{2}$ for the expectations of *a*_1_ (or *a*_2_) and ${a_{1}^{2}}$ then finally yields

(10) $$  E\left[\!|A_{N}(f)|^{2}\right] = N \bar{\bar{{a}}}^{2} + N(N-1) \bar{a}^{2} \text{sinc}^{2}(\pi f T),  $$

with sinc(*x*)= sin(*x*)/*x*.

The expected power spectral density of the EMG can be calculated from the energy spectral density of a finite sequence of MUAPs, |*S*(*f*)|^2^, as

(11) $$ P_{s}(f) = {\lim}_{\textit{T}\to\infty} \frac{E[|S(f)|^{2}]}{T} = |H(f)|^{2} \cdot {\lim}_{\textit{T}\to\infty} \frac{E[|A_{N}(f)|^{2}]}{T}.  $$

If the time *T* is sufficiently long, the second term in Eq. (10) is negligible. Moreover, *N* can be replaced by *ρ*_0_*T*, where *ρ*_0_ is the mean MUAP rate. So we finally get

(12) $$  P_{s}(f) = \bar{\bar{{a}}}^{2} \rho_{0} |H(f)|^{2}.  $$

Note that *P*_*s*_(*f*) is to be understood as a two-sided power spectral density. Thus, the total power is obtained by integrating over positive as well as negative frequencies, yielding ${\bar {\bar {{a}}}}^{2} \rho _{0} \|{H}\|^{2}_{2}$, where

(13) $$  \|H\|_{2} = \sqrt{\int_{-\infty}^{\infty} |H(f)|^{2} df }  $$

is the *L*^2^ norm of *H*(*f*). As required by Parseval’s theorem (see, e.g., [9]), this result is consistent with a previous time-domain analysis [5], in which the variance of the EMG was shown to be ${\bar {\bar {{a}}}}^{2} \rho _{0} \|{H}\|^{2}_{2}$, where

(14) $$  \|h\|_{2} = \sqrt{\int_{-\infty}^{\infty} h(t)^{2} dt }  $$

is the *L*^2^ norm of *h*(*t*).

In practice, the power spectrum has to be estimated from a finite sample of the EMG, and so the question arises whether it is justified to neglect the second term on the right-hand side of Eq. (10). As $\bar {a}$ and $ {\bar {\bar {{a}}}}$ typically have about the same value, the condition to be fulfilled is *N*/(*πfT*)^2^≪1. With *N*=*ρ*_0_*T*, as already assumed above, the condition can be rewritten as *f*^2^≫*ρ*_0_/(*π*^2^*T*). Making the more or less realistic assumptions *ρ*_0_=1000 s ^−1^ (see, e.g., [4]) and *T*=10 s, the requirement can be approximated as *f*^2^≫10 s ^−2^. This simple estimation suggests that proportionality between |*H*(*f*)|^2^ and power spectrum of the EMG can be expected only at sufficiently high frequencies. In this article we generally choose a lower frequency limit of 10 Hz.

### Optimization problem

Conceptually, the goal is to solve Eq. (2) for *R*(*f*). However, this cannot be accomplished in a direct way because only the magnitude of *H*(*f*) can be estimated from the EMG. We overcome the problem of incomplete information by assuming that *R*(*f*) is a well-defined function with some unknown parameters. Taking the square root of the estimated EMG power spectral density as an estimate of the magnitude of the Fourier transformed MUAP (denoted as $|\bar {H}(f)|$ in what follows), the magnitude of the Fourier transformed VEMP can be predicted as

(15) $$  |\hat{V}(f)| = |\bar{H}(f)| \cdot |R(f)|.  $$

The function *R*(*f*) can be optimized, then, by relating this prediction to the VEMP actually measured. More precisely, optimal parameter values for *R*(*f*) can be determined by minimizing a cost function of the form

(16) $$  Q_{\kappa,\lambda} = \int_{f_{\text{min}}}^{f_{\text{max}}} \left| \left(\frac{|\bar{V}(f)|}{\|\bar{V}\|_{*}}\right)^{\kappa} - \left(\frac{|\hat{V}(f)|}{\|\hat{V}\|_{*}}\right)^{\kappa} \right|^{\lambda/2} df,  $$

where $\bar {V}(f)$ is the Fourier transform of the measured VEMP, and ∥.∥_∗_ is a norm defined as

(17) $$ \|X\|_{*} = \sqrt{\int_{f_{\text{min}}}^{f_{\text{max}}} |X(f)|^{2} df }.  $$

For symmetry reasons there is no need to consider negative frequencies. A lower frequency limit, *f*_min_, is defined to avoid the problem considered at the end of the previous subsection. Defining an upper frequency limit, *f*_max_, may be useful to exclude noise outside the frequency range of interest. The normalization overcomes the problem that $|\hat {V}(f)|$ is arbitrarily scaled. The nature of the cost function can be varied by means of the parameters *κ* and *λ*, which are assumed to be integers. Extensive simulations (presented below) suggest that, all in all, the cost function *Q*_2,1_ is a good default choice.

### Modelling the rate modulation

The function to be optimized, *R*(*f*), is conveniently defined as the Fourier transform of a rate modulation designed in the time-domain. As in a previous article [6], the function

(18) $$  r(t) = \sum_{k=1}^{K} r_{k} \exp\left(-\frac{1}{2}\left(\frac{t-t_{k}}{\tau_{k}}\right)^{2} \right)  $$

is chosen, which means that the rate modulation is modelled as a sequence of *K* Gaussian pulses. The parameters *t*_*k*_, *r*_*k*_, and *τ*_*k*_ determine the time, the amplitude, and the width of the *k*-th peak (1≤*k*≤*K*). A positive value of *r*_*k*_ indicates excitation, whereas a negative value indicates inhibition. The Fourier transform of *r*(*t*) is

(19) $$  R(f) = \sqrt{2\pi} \sum_{k=1}^{K} r_{k} \tau_{k} \exp\left(-2\pi^{2} f^{2} {\tau_{k}^{2}} - \mathfrak{i} 2\pi f t_{k} \right).  $$

This function has more parameters than can be determined by minimizing the right-hand side of Eq. (16). The reason is that *r*(*t*) is in this context indistinguishable from *c*·*r*(*t*−*t*_0_), assuming *c*≠0. We fix this problem by setting *r*_1_=−1 and *t*_1_=0. Thus, in the simplest case (*K*=1) there is only a single parameter to be optimized: *τ*_1_.

**Fig. 1:**
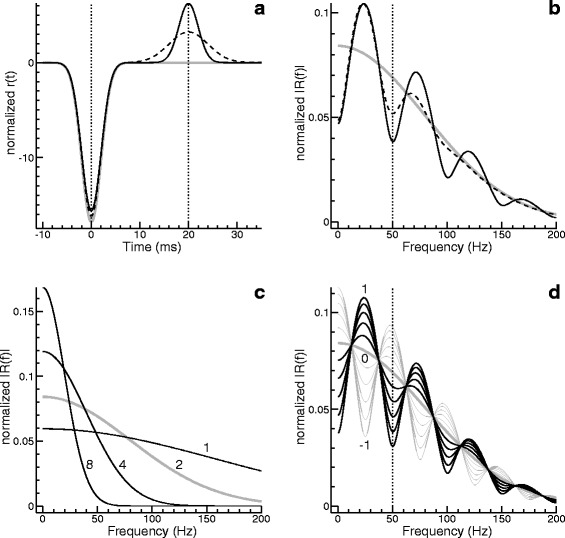
Examples of rate modulation functions and their frequency-domain representations. **a** A single-peaked function (grey curve in the background) and two double-peaked functions. **b** Modulus of the Fourier transform of the functions in (a). **c** Modulus of the Fourier transform of single-peaked rate modulations varying in the parameter *τ* (1, 2, 4 or 8 ms). **d** Modulus of the Fourier transform of double-peaked rate modulations with *t*_2_=20 ms, *τ*_1_=*τ*_2_=2 ms, *r*_1_=−1 and *r*_2_ varying in steps of 0.2 from -1 to 1

Examplary rate modulation functions are shown in Fig. 1. To facilitate the comparison, the functions were normalized using the *L*^2^ norm. The squared *L*^2^ norm of *r* (respectively *R*) can be shown to be

(20) $$ \|r\|_{2}^{2} = \|R\|_{2}^{2} = \sqrt{2\pi} \sum_{k=1}^{K} \sum_{l=1}^{K} r_{k} r_{l} \frac{\tau_{k} \tau_{l}}{\sqrt{{\tau_{k}^{2}}+{\tau_{l}^{2}}}} \exp\left(-\frac{(t_{k}-t_{l})^{2}}{2({\tau_{k}^{2}}+{\tau_{l}^{2}})} \right).  $$

The three rate modulation functions presented in Fig. 1a agree regarding the first peak, for which we assumed *r*_1_=−1, *t*_1_=0, and *τ*_1_=2 ms (the apparent minor differences between the curves go back to the normalization). While the grey curve shows only this one peak, the other two curves show a second peak of opposite polarity, centered at *t*_2_=20 ms. In the case of the solid black curve we assumed *τ*_2_=*τ*_1_, whereas in the case of the dashed curve we assumed *τ*_2_=2*τ*_1_. Moreover, as to the peak amplitude we assumed *r*_2_*τ*_2_=0.8 ms, which ensures that the area under the second peak is the same for the two cases.

The differences in the time domain are paralleled by characteristic differences in the frequency domain (Fig. 1b). For the single-peaked rate modulation, the magnitude of the Fourier transform is bell-shaped (grey curve), whereas the other two curves appear to oscillate around this curve. Analytical considerations help to understand this observation. For *K*=2 and *τ*_1_=*τ*_2_=*τ* we get

(21) $$  |R(f)| = \sqrt{2\pi} \tau \exp\left(-2\pi^{2} f^{2} \tau^{2}\right) \sqrt{{r_{1}^{2}}+{r_{2}^{2}}+2 r_{1} r_{2} \cos\Big(2\pi f (t_{2}-t_{1}) \Big) }.  $$

The exponential term clearly explains the bell shape of the grey curve, whereas the cosine term explains the oscillations of the other two curves. Equation (21) predicts a spectral minimum at *f*=1/(*t*_2_−*t*_1_), which is 50 Hz in the present example. This minimum is a potentially important fingerprint when analysing and interpreting real data. Hence, it is noteworthy that the minimum is found also in the dashed curve, although the assumption *τ*_1_=*τ*_2_, on which Eq. (21) is based, is not fulfilled.

Figure 1c illustrates how the magnitude of the Fourier transform depends on the parameter *τ*, which was varied between 1 and 8 ms. All curves refer to a single-peaked rate modulation; the grey curve (*τ*=2 ms) is identical with the grey curve in Fig. 1b. A comparison of the curves for *τ*=2 ms and *τ*=4 ms gives a hint as to why the oscillation of the dashed curve in Fig. 1b fades more rapidly with increasing frequency than does the oscillation of the solid black curve: The broader the second peak is in the time domain, the less does it contribute to higher frequencies.

In Fig. 1d, the parameter *r*_2_ was systematically varied between -1 and 1, whereas *τ*_2_ was kept constant at 2 ms. The thick grey curve, representing the case *r*_2_=0, is again identical with the bell-shaped grey curve in Fig. 1b, whereas the black and the thin grey curves were obtained for *r*_2_>0 and *r*_2_<0, respectively. As to be expected in view of Eq. (21), the parameter *r*_2_ determines magnitude and polarity of the deviation from the bell-shaped “baseline”.

### VEMP deconvolution

The optimization procedure described above was devised to deal with the problem that only the magnitude of *H*(*f*) can be estimated from the EMG. Having completed the parameter optimization, the missing phase information can be obtained as well, if desired. According to Eq. (2), *H*(*f*) could be estimated as $\bar {V}(f)/R(f)$, except for a constant factor. But such a direct approach might lead to problems at frequencies where |*R*(*f*)| is close to zero, because noise would be enormously amplified. The problem can be avoided by using the estimator

(22) $$  \hat{H}(f) = \frac{1}{1+\epsilon R_{\max}^{2} /| R(f)|^{2}}\cdot \frac{\bar{V}(f)}{R(f)},  $$

where *R*_max_ is the maximum value of |*R*(*f*)| and *ε*≪1 is a positive real number. The first factor has basically no effect for $|R(f)|^{2}\gg \epsilon R_{\max }^{2}$, whereas for $|R(f)|^{2}\ll \epsilon R_{\max }^{2}$ it causes $|\hat {H}(f)|$ to have a value close to zero. Lütkenhöner and Basel [6] showed that this kind of regularization can be understood as optimal (Wiener) filtering. In this article we defined *ε* as 10^−2^.

### Non-uniqueness

The setup of the optimization procedure already accounted for the fact that the rate modulation can only be determined up to an unknown scale factor and that any time shift of the rate modulation can be compensated for by a reverse time shift of the MUAP. But there is yet another kind of non-uniqueness inherent to the inverse problem considered here: Time reversal of the rate modulation function, which in the frequency domain corresponds to substituting *R*(*f*) by its complex conjugate, ${R}^{*}(f)=|R(f)|e^{-\mathfrak {i}\arg (R(f))}$, has no effect on the cost function defined in Eq. (16). Using *R*^∗^(*f*) rather than *R*(*f*) in Eq. (22) yields $\hat {H}_{{R}^{*}}(f)=\hat {H}(f)e^{2\mathfrak {i}\arg (R(f))}$. The fact that $\hat {H}(f)$ and $\hat {H}_{{R}^{*}}(f)$ usually differ fundamentally could help to resolve the ambiguity related to time reversal, provided that a rough idea about the phase of *H*(*f*) is available. For example, it might be reasonable to assume that *H*(*f*) more likely resembles a minimum rather than a maximum phase filter.

## Simulation results

### Simple Gaussian model

**Fig. 2:**
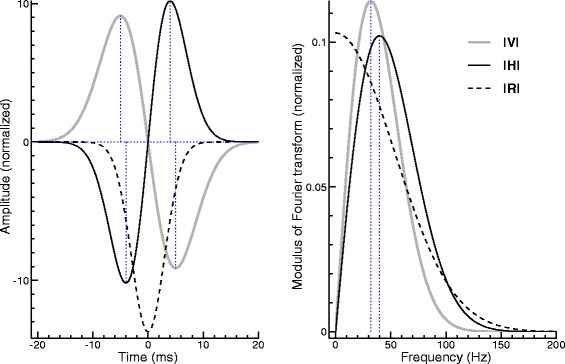
Simple Gaussian model in the time domain (*left*) and the frequency domain (*right*). Convolution of the rate modulation (*dotted curve*) with the MUAP (*solid black curve*) yields the VEMP (*grey curve*)

In the simplest case, the rate modulation specified in Eq. (18) consists of a single component, and only the parameter *τ*_1_=*τ* has to be estimated from the data. Such a situation is considered in Fig. 2. In the time-domain representation on the left, the rate modulation *r*(*t*) is shown as a dashed curve, the MUAP *h*(*t*) as a black solid curve, and the VEMP *v*(*t*) as a gray curve. The MUAP has the shape proposed by Wit and Kingma [4]. With regard to Eq. (24), which can be found below in the section “Methods”, this means *θ*_1_=*θ*_2_=*θ*. Lütkenhöner and Basel [10] showed that the VEMP obtained by convolving such a MUAP with a Gaussian pulse is described by the same function as the MUAP itself, except that *θ* has to be replaced by

(23) $$  \theta_{v}=\sqrt{\theta^{2}+\tau^{2}}.  $$

In the present example, *τ* and *θ* have the values 3 and 4 ms, respectively, so that *θ*_*v*_ is 5 ms. The extrema of *h*(*t*) and *v*(*t*) are found at the times ±*θ* and ±*θ*_*v*_, respectively (dotted vertical lines).

Calculating the modulus of the Fourier transform yields the curves shown on the right of Fig. 2. In view of the above time-domain considerations it is not surprising that the curves for MUAP (black) and VEMP (gray) are similar. The differences between these curves can be exploited to draw conclusions as to the rate modulation. This is most easily done by comparing the locations of the spectral maxima, which, for the situation considered here, are found at the frequencies 1/(2*πθ*) and 1/(2*πθ*_*v*_), respectively [10]. Numerical evaluation yields 39.8 and 31.8 Hz, respectively (dotted vertical lines). By reversing the line of thought, the frequencies of experimentally determined spectral maxima of MUAP and VEMP can be converted into estimates of *θ* and *θ*_*v*_, and *τ* can subsequently be calculated using Eq. (23).

But relying on a single feature is generally not to be recommended when dealing with real data. A more faithful estimate of *τ* is obtained by minimizing a cost function as in Eq. (16). Extensive simulations were performed to answer the question as to what cost function is the most advantageous one. As distinct from Fig. 2, the rate modulation was scaled so that the peak value was *r*_1_(0)=−0.5. Moreover, the MUAP rate *ρ*_0_ was set to 1000 s ^−1^, 100 VEMPs were elicited at a rate of 4 s ^−1^, and the simulated EMG was sampled at a rate of 2000 s ^−1^. All in all, 10,000 Monte Carlo experiments were carried out, and in each case the parameter *τ* was optimized using various cost functions. Thus, in the end, 10,000 estimates of *τ* were obtained for each of the investigated cost functions. Table 1 summarizes the results of a statistical analysis of these data. Each column corresponds to a specific cost function. A comparison of the median and the mean of the estimated *τ* with the true value (3 ms) suggests that the least biased estimate is obtained by minimizing the cost function *Q*_2,1_. On the other hand, minimization of the cost function *Q*_1,2_ resulted in the smallest standard deviation (SD) as well as the smallest root-mean-square deviation (RMSD) between estimated and true *τ*.

**Table 1:** Estimation of the parameter *τ* of the simple Gaussian model

	*Q*_1,1_	*Q*_1,2_	*Q*_1,4_	*Q*_2,1_	*Q*_2,2_	*Q*_2,4_
Median	2.47	2.61	2.70	**2.80**	2.70	2.61
Mean	2.49	2.65	2.71	**2.83**	2.74	2.62
SD	0.62	**0.59**	0.68	0.72	0.83	0.97
RMSD	0.80	**0.69**	0.74	0.74	0.87	1.04

The parameter estimation was performed using six different cost functions. Based on 10,000 simulated experiments, the median, the mean, the standard deviation (SD) and the root-mean-square deviation (RMSD) of the estimated *τ* were estimated (true value: 3 ms). The optimal result for each measure, which is the least biased median or mean and the smallest SD or RMSD, is shown in bold

### More sophisticated model

**Fig. 3:**
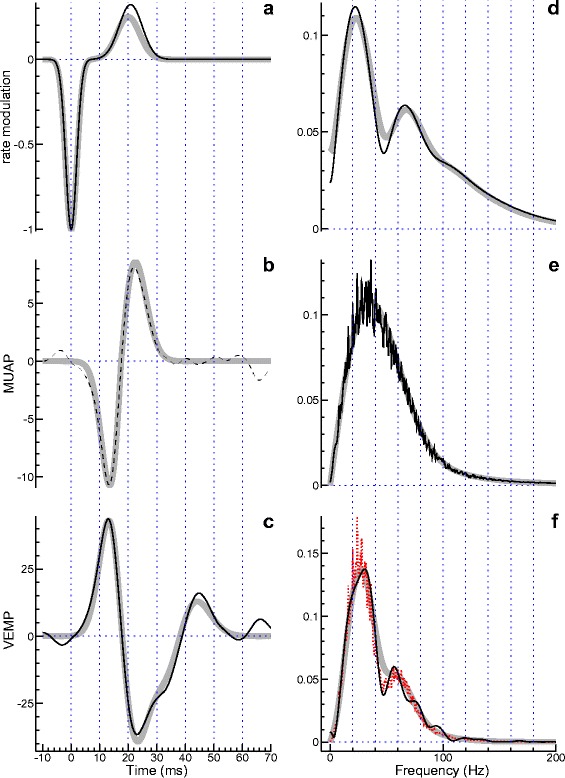
Simulation using a model with double-peaked rate modulation and slightly asymmetric MUAP. The theoretical functions are shown as grey curves in the background. The time domain is considered on the left, the frequency domain on the right. The black curves in **c** and **e** were obtained by analysing Monte Carlo simulated data. They show the estimated VEMP and the square root of the normalized EMG power spectrum, respectively. The latter is used as a surrogate for the modulus of the Fourier transform of the MUAP. The rate modulation shown in **a** and **d** was estimated by minimizing the cost function *Q*_2,1_ (see Eq. (16)). Loosely speaking, the rate modulation was chosen so that the frequency-domain representation of the predicted VEMP (shown as a dotted (red) curve in **f**) optimally corresponded to the modulus of the Fourier transform of the measured VEMP (black curve). Deconvolution of the measured VEMP with the estimated rate modulation yielded an estimate of the MUAP (dashed curve in **b**)

While the simple Gaussian model appears to be ideally suited for getting a first understanding of the relationship between MUAP and VEMP, the underlying assumptions are relatively special. Therefore another, more sophisticated model will be investigated now. The model itself is illustrated by the thick grey curves in the background of Fig. 3. The two-component rate modulation shown in Fig. 3a was calculated using Eq. (18), with *r*_1_=−1, *r*_2_=0.25, *τ*_1_=2 ms, *τ*_2_=4 ms, *t*_1_=0, and *t*_2_=20 ms. The *L*^2^-normalized MUAP function shown in Fig. 3b was calculated using Eq. (24), which can be found below in the section “Methods” (the parameters were *θ*_1_=4 ms and *θ*_2_=5 ms; moreover, the curve was time-shifted by 17.5 ms). Convolution of rate modulation and MUAP yields the VEMP shown as a grey curve in the background of Fig. 3c. The gray curves on the right side of the figure show the modulus of the Fourier transform for the corresponding curves on the left side.

The grey curves in Fig. 3 visualize theoretical functions which are, of course, not available when dealing with real data. Instead, the analysis has to begin with two curves that are generally quite noisy: the estimated VEMP and the estimated power spectral density of the EMG. Here we consider the square root of the latter, which serves as a surrogate for the modulus of the Fourier transform of the MUAP. The examples shown as black curves in Fig. 3c and 3e, respectively, were obtained by Monte Carlo simulation. As in the simulations before, the MUAP rate *ρ*_0_ was set to 1000 s ^−1^, the data was sampled at a rate of 2000 s ^−1^, and stimuli were presented at a rate of 4 s ^−1^. The number of stimulus presentations was raised to 200, though. In accordance with the Theory section of the article, where it was explained that the EMG power spectrum is basically proportional to the energy spectral density of the MUAP, the grey curve in the background of Fig. 3e appears, indeed, as a smoothed version of the black curve.

What remains to be done now is to choose a rate modulation so that, in the frequency-domain, multiplication with the estimate obtained for the MUAP (square root of the EMG power) optimally fits the observed VEMP. The frequency-domain representation of the VEMP is shown as a black curve in Fig. 3f, whereas the best fitting model (the one that minimizes the cost function *Q*_2,1_) is represented by the dotted (red) curve. The corresponding rate modulation, represented by the black curve in Fig. 3a, is in good agreement with the theoretical counterpart (grey curve).

Estimation of the MUAP function *h*(*t*) is optional, but can help to check the plausibility of the rate modulation obtained. The dashed curve in Fig. 3b shows the Fourier transform of $\hat {H}(f)$ as calculated using Eq. (22). The curve apparently agrees well with the theoretical counterpart (grey curve).

To get a more representative view, the Monte Carlo experiment considered in Fig. 3 was repeated 10,000 times, and as in the case of the simple Gaussian model considered before, the parameter estimation was done with different cost functions. A statistical evaluation of the results is provided in Table 2. The table is analogous to the previous one, except that there are four parameters rather than one. The optimal result in each row (median or mean showing the smallest deviation from the true value; smallest SD or RMSD) is shown in bold. In the majority of cases the optimal result was obtained for the cost function *Q*_2,1_, and if another cost function performed better, the advantage was generally small (even marginal in the case of the parameter *r*_2_). Thus, all in all, the cost function *Q*_2,1_ appears to be the most favorable choice.

**Table 2:** Parameter estimation for the two-component model

		Cost function					
		*Q*_1,1_	*Q*_1,2_	*Q*_1,4_	*Q*_2,1_	*Q*_2,2_	*Q*_2,4_
*τ*_1_	median	1.77	1.82	1.85	**1.92**	1.81	1.32
(ms)	mean	1.78	1.84	1.86	**1.94**	1.86	1.59
	SD	**0.20**	0.24	0.41	0.28	0.51	0.66
	RMSD	0.29	0.29	0.43	**0.28**	0.53	0.78
*τ*_2_	median	4.23	3.86	3.61	**4.04**	3.76	3.14
(ms)	mean	4.38	4.17	3.88	4.15	**3.92**	3.54
	SD	1.21	1.76	2.09	**1.14**	2.24	2.62
	RMSD	1.27	1.77	2.09	**1.15**	2.24	2.66
*r*_2_	median	**0.25**	0.26	0.26	0.25	0.26	0.21
	mean	**0.25**	0.27	0.29	0.25	0.30	0.28
	SD	**0.08**	0.09	0.13	0.09	0.16	0.18
	RMSD	**0.08**	0.09	0.14	0.09	0.17	0.18
*t*_2_	median	19.87	19.90	19.86	**19.98**	19.88	19.85
(ms)	mean	19.82	19.80	19.76	**19.99**	19.82	19.83
	SD	1.62	1.65	1.61	**1.52**	1.64	1.85
	RMSD	1.63	1.66	1.63	**1.52**	1.65	1.86

The table is organized in the same way as Table 1, except that there are four parameters now (true values: *τ*_1_=2 ms, *τ*_2_=4 ms, *r*_1_=0.25, *t*_2_=20 ms). The optimal result for each measure, which is the least biased median or mean and the smallest SD or RMSD, is shown in bold

## Analysis of exemplary real data

**Fig. 4:**
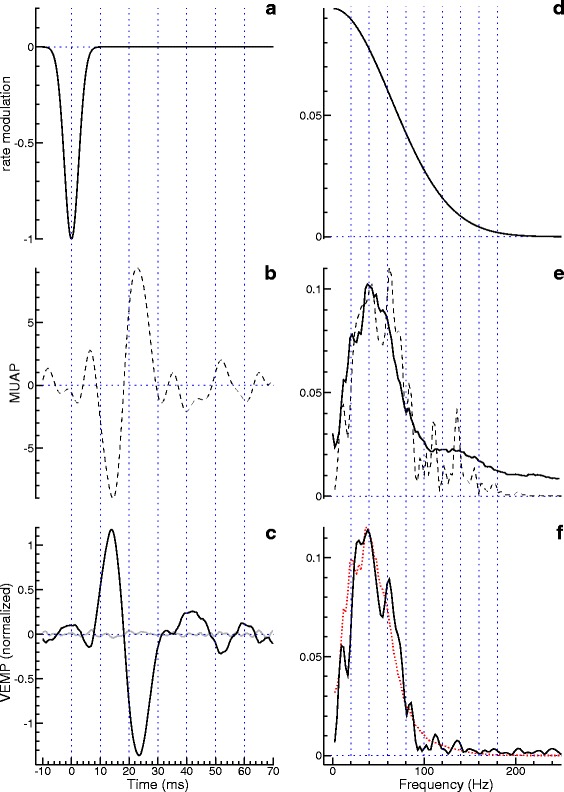
Real-data example of a single-component VEMP. The figure is organized in basically the same way as Fig. 3. The time domain is considered on the left, the frequency domain on the right. The measured data are represented by the black curves in **c** and **e**, which show the VEMP and the square root of the EMG power spectrum, respectively. The rate modulation shown in **a** and **d** was optimized so that the frequency-domain representation of the predicted VEMP, shown as a dotted (red) curve in **f**, optimally corresponded to the modulus of the Fourier transform of the measured VEMP (black curve in **f**). Deconvolution of the measured VEMP with the estimated rate modulation yielded an estimate of the MUAP (dashed curve in **b**)

To investigate the applicability of the proposed method under real-world conditions, two exemplary datasets were investigated. The data as well as its model-based interpretation are presented analogously to the simulation results shown in Fig. 3. In the first example, the VEMP (Fig. 4c) shows prominent peaks with latencies around 13 and 23 ms. Considering their polarity they are called p13 and n23. Visual inspection of the curve gives no indication that this standard component of the VEMP is followed by a second component of significant amplitude. The analysis was therefore done with a one-component rate modulation (Fig. 4a). As in the simulations presented before, this function was iteratively adjusted so that the modulus of its Fourier transform (Fig. 4d) multiplied by the square root of the EMG power spectrum estimated from the data (black curve in Fig. 4e) approximated the modulus of the Fourier transformed VEMP (solid curve in Fig. 4f) in the best possible way. The dotted curve in the latter panel, representing the modelling result, demonstrates that the data is explained quite well by our simple model, althought it has just one free parameter, *τ*_1_ (for which we dermined the value 2.5 ms). The optional estimation of the MUAP function yielded the dashed curve in Fig. 4b. Fourier transformation of this curve resulted in the dashed curve in Fig. 4e, which is roughly consistent with the black curve in that panel. The deviations at higher frequencies are caused by the regularization factor in Eq. (22). A convolution of the rate modulation (Fig. 4a) with the estimated MUAP (Fig. 4b) yields a curve that almost coincides with the measured VEMP (difference shown as a grey curve in Fig. 4c). This good agreement was to be expected, because the convolution basically inverts the estimation of the MUAP.

**Fig. 5:**
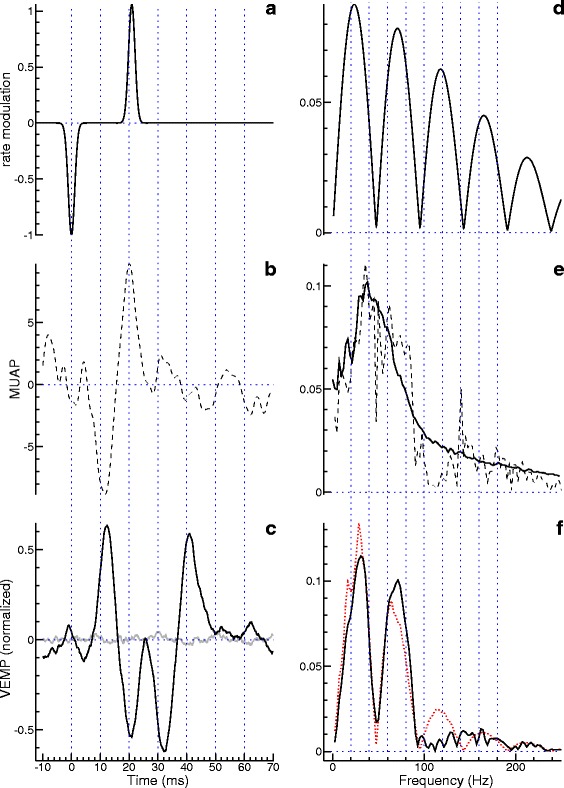
Real-data example of a two-component VEMP. The figure is organized in exactly the same way as Fig. 4

In the second example, the VEMP (Fig. 5c) is evidently composed of two components: The first component, with the peaks p13 and n23, is followed by a second component with peaks around 34 and 44 ms. Considering their polarity they are called n34 and p44. Colebatch et al. [11] presumed that the second component is of cochlear rather than vestibular origin. Figure 5a shows the rate modulation determined for this example. To keep the number of parameters at a minimum, the two peaks of the rate modulation were assumed to have the same width, which in terms of Eq. (18) means *τ*_1_=*τ*_2_=*τ*. Optimization of the three remaining model parameters yielded *τ*=1.1 ms, *r*_2_=1.1, and *t*_2_=20.9 ms. Figure 5f shows a good agreement between data (solid curve) and model prediction (dotted curve). Thus, there seems to be no reason to use a more elaborate model. The improvement obtained by dropping the assumption *τ*_1_=*τ*_2_ (not shown in the figure) was indeed marginal. As in the previous example, the optional calculation of the MUAP yielded a roughly biphasic curve (Fig. 5b), the Fourier transform of which is shown as a dashed curve in Fig. 5e. The VEMP predicted by convolving the estimated MUAP with the estimated rate modulation is again almost identical with the measured VEMP (difference shown as a grey curve in Fig. 5c).

## Discussion and conclusions

This study started from the conjecture that the differences in the spectral densities of VEMP and EMG represent a signature which, if interpreted appropriately, can tell something about the generation of the VEMP. Model simulations building on previous theoretical work [4, 5, 10] allowed us to make predictions about this signature. Our current understanding is that the VEMP represents a brief reduction of the firing probability of the motor units. To implement this idea in the convolution model given by Eq. (1), the rate modulation is defined as a negative pulse. The width of the pulse can principally be estimated by comparing the locations of the spectral maxima of VEMP and EMG: the more they differ, the broader the peak (Fig. 1c). But when working with real data such as those presented in Fig. 4, noise typically prevents a precise estimation of spectral maxima. The problem can be overcome by resorting to a parameter optimization approach which accounts for the whole (or nearly the whole) spectrum. This way we derived the rate modulation shown in Fig. 4a. For a Gaussian function with the standard deviation *τ*, the full width at half maximum is $2\sqrt {2\ln 2} \tau \approx 2.355 \tau $. Inserting the estimated *τ* we get 5.9 ms, which is consistent with Colebatch and Rothwell [12]: In a study of single motor units they found inhibition windows with a duration between 2 and 8 ms (mean 3.6 ms).

A second VEMP component, which follows roughly 20 ms after the first one and has opposite polarity, is found in 55 % [13] to 76 % [14] of healthy subjects. Because this component does not depend on the integrity of the vestibular nerve, it is generally assumed to be of cochlear rather than vestibular origin [11]. But the component was identified also in deaf ears, which could be indicative of a dual origin: cochlear as well as vestibular [13]. No matter what the origin of the second component is, the analysis of a VEMP consisting of two components requires to choose a rate modulation with two peaks. In that case, the spectral density of the VEMP differs from the spectral density of the EMG by a more or less pronounced spectral minimum. This kind of signature is so salient in Fig. 5f that the evaluation can be done just by visual inspection. The location of the spectral minimum is easy to interpret, because it corresponds to the reciprocal of the latency difference between the two VEMP components. Other frequency-domain features are more difficult to understand. Thus, a parameter optimization has to be performed to explain given data in terms of a model. In the example presented in Fig. 5, the assumption of a two-component rate modulation was indeed suitable to explain the data. It was even possible to reduce the number of parameters by assuming that the two peaks of the rate modulation have the same width.

A key feature of the proposed method is that the rate modulation is characterized in terms of just a few parameters, whereas no assumptions whatsoever are made about the MUAP. The latter is considered as an arbitrary function to be estimated from the data (more precisely, only the modulus of the Fourier transform is relevant). Thus, the finding that the estimated MUAP (Figs. 4b and 5b) is roughly biphasic, in good agreement with the theoretical functions used in the model simulations, does not reflect assumptions inherent to the model, but is based on the measured data. The reason why the rate modulation rather than the MUAP was parametrized (in theory, the latter possibility would work equally well) is that parameters closely related to the questions of interest can be chosen. If the VEMP has two components, the questions concern the latency difference as well as the amplitude ratio of the components, and the half width of each component. Inhibitory and excitatory rate modulations are distinguished by the sign, but apart from that they are handled in the same way. Although the method cannot be used to confirm the generally accepted opinion that the first VEMP component (p13-n23) is of an inhibitory nature, it is possible to relate the “polarity” (excitatory versus inhibitory) of any other component to that of the first component.

A drawback of the normalization in Eq. (16) is that the estimated functions are unscaled. In this respect the present approach is clearly inferior to a method developed by Lütkenhöner and Basel [6], where the deconvolution algorithm exploits the relationship between VEMP and associated variance modulation. But that method is applicable only in well-chosen cases, because a variance modulation with a sufficient signal-to-noise ratio is rather the exception. The present approach, by contrast, promises to be workable whenever an acceptable VEMP was recorded, because this normally means that the EMG power spectrum can be estimated reasonably well, too. Considering the fact that both deconvolution approaches have advantages and disadvantages, the question arises as to whether they can be combined. This should indeed be possible. The general idea is that the unscaled functions obtained with the method proposed here are scaled using the previously developed algorithm. Conceptually, given unscaled estimates of rate modulation and MUAP, a simulation as presented in Fig. 3 could be run, provided that suitable assumptions are made about what is unknown. Detailed knowledge of the statistical distribution of the MUAP amplitudes is not essential, because a normalization of the EMG (such that it has unit variance) prior to the estimation of the VEMP basically eliminates the dependence of the VEMP on the MUAP amplitudes [5]. In essence, only two unknown parameters remain: the mean MUAP rate and the scaling factor for the rate modulation. These two parameters could be optimized by comparing the variance modulation estimated from simulated EMG data with the variance modulation derived from the experimental data. Even if the signal-to-noise ratio of the latter is too bad for a stand-alone deconvolution as proposed in our previous article [6], there may be enough information for optimizing only two parameters. This consideration shows that the deconvolution method developed in this study could eventually become a key component of a more comprehensive deconvolution approach that simultaneously works on VEMP, associated variance modulation, and power spectrum of the EMG.

## Methods

### Monte Carlo experiments

A synthetic EMG was calculated using Eq. (3). As to the definition of the MUAP function *h*(*t*), we basically followed Wit and Kingma [4], who suggested to use the first derivative of a Gaussian. Such a function is symmetric in the sense that its shape is invariant under time reversal (apart from the sign). However, to be more realistic, it could be useful to allow for some degree of asymmetry, and therefore we defined the MUAP function as follows:

(24) $$  h(t) = \frac{2\pi^{-1/4}}{\sqrt{\theta_{1}^{-1}+\theta_{2}^{-1}}} \left\{ \begin{array}{r@{\quad\text{if}\quad}l} t \theta_{1}^{-2} \exp\left(-t^{2}\theta_{1}^{-2}/2\right) & t<0\\ t \theta_{2}^{-2} \exp\left(-t^{2}\theta_{2}^{-2}/2\right) & t\ge{0} \end{array} \right.  $$

**Fig. 6:**
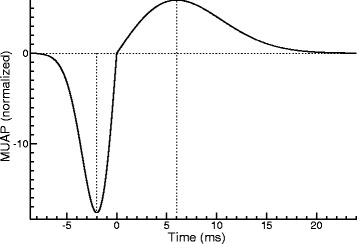
The MUAP function defined in Eq. (24) for *θ*_1_=2 ms and *θ*_2_=6 ms

This function has an *L*^2^ norm of one and mean zero. It is continuous at *t*=0, but only for *θ*_1_=*θ*_2_ it is continuously differentiable at this point. The latter requirement corresponds to the original proposal by Wit and Kingma [4]. Figure 6 shows the graph of *h*(*t*) for *θ*_1_=2 ms and *θ*_2_=6 ms. The function reaches a minimum at *t*=−*θ*_1_ and a maximum at *t*=*θ*_2_ (see dotted vertical lines).

The application of Eq. (3) requires that random numbers *a*_*n*_ and *t*_*n*_ (1≤*n*≤*N*) are generated. Strictly speaking, the total number of contributing MUAPs, *N*, is a random number as well. As to the MUAP occurrence times *t*_*n*_, the task was accomplished by considering the MUAP generation as a time-dependent Poisson process (see, e.g., [15]). More specifically, the mean number of MUAPs occurring between *t* and *t*+*Δt* was assumed to be *ρ*(*t*)*Δt*, where *ρ*(*t*) is a rate function; the Matlab function POISSRND converted the mean number into a random number of MUAPs (mostly 0 or 1, provided that *Δt* is sufficiently small; the simulations presented here were performed with *Δt*=0.5 ms). To generate all the required MUAP occurrence times, the time range of interest (with some extra time on both sides, to avoid edge effects) was sampled at intervals of *Δt*, and the idea of a Poisson process was successively applied to each sampling time. The amplitude values associated with the occurrence times were drawn from a gamma distribution with shape parameter 2, as already suggested by Wit and Kingma [4]. For the sake of convenience, the value 1/2 was chosen for the scale parameter of the distribution, which has the consequence that the mean amplitude is $\bar {a}=1$. Gamma distributed random numbers were generated using the Matlab function GAMRND.

What remains to be done is to specify the rate function *ρ*(*t*). This function is closely related to one of the key quantities of the present study: By definition of the rate modulation *r*(*t*), the rate is

(25) $$  \rho(t) = \rho_{0} \cdot \big(1+r(t)\big),  $$

where *ρ*_0_ denotes, as in the context of Eq. (12), the mean MUAP rate of the undisturbed EMG (no VEMP eliciting stimulus). To simulate a typical VEMP experiment, the stimulus was assumed to be presented at intervals of 250 ms, and a corresponding periodicity was implemented also into the rate *ρ*(*t*).

The Monte Carlo simulations were complemented by numerical calculations based on Eq. (1). The constant factor in that equation can be expressed as

(26) $$  c_{v} = \bar{a} \rho_{0}  $$

under the conditions considered here [5]. The convolution integral was calculated using the Matlab function CONV.

### Exemplary real data

Two exemplary sets of real data were taken from an archive compiled by Lütkenhöner et al. [16], which comprises data from patients who underwent VEMP testing as part of a clinical evaluation of their dizziness symptoms. In brief, VEMPs were elicited by 500-Hz Gaussian tone pulses with a full width at half maximum of 4 ms, which were presented at a rate of 4/s (peak-equivalent sound pressure level of 107 dB). The electromyogram, recorded from the sternocleidomastoid muscle, was continuously digitized at a rate of 10 kHz. Each data set comprises 200 stimulus presentations.

### Estimation of the rate modulation from given data

**Fig. 7:**
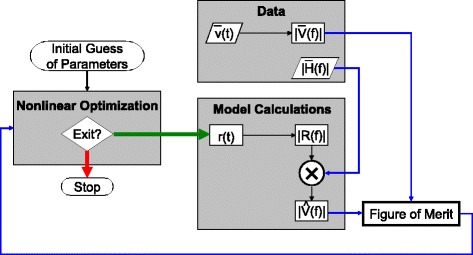
Sketch of the algorithm. A nonlinear optimization routine adjusts the parameters of the rate modulation, *r*(*t*), in such a way that, as to the magnitude of the Fourier transform, the predicted VEMP, $\hat {V}$, and the measured VEMP, $\bar {V}$, optimally match. The prediction is based on the idea that the VEMP can be calculated by convolving rate modulation and MUAP and that the magnitude of the Fourier transform of the latter basically corresponds to the square root of the EMG power spectrum (estimate denoted as $|\bar {H}(f)|$)

Simulated as well as real data were analysed using custom Matlab scripts. The estimation of the rate modulation from given data requires determining optimal values for a limited number of parameters. This inherently nonlinear problem was solved using the Matlab function FMINSEARCH, which is an implementation of the simplex search method of Lagarias et al. [17]: a direct search method which gets along without calculating numerical or analytic gradients. Figure 7 schematically illustrates how the nonlinear optimization routine drives the overall algorithm. The optimization is started by providing an initial guess of the parameters, which are, then, iteratively improved until a predefined exit condition is fulfilled. It is convenient to define the rate modulation in the time domain, although the algorithm itself works with the magnitude of the Fourier transform. The number of parameters should be restricted to a minimum. A single parameter (defining the duration of the rate modulation) can be sufficient for VEMPs showing no clear indication of a second component, whereas for VEMPs consisting of two components, three or four parameters can be sufficient (defining the amplitude ratio and the time lag of the two rate modulations as well as their durations; it may be possible to assume that the latter two parameters are identical).

The data required by the algorithm consist of the measured VEMP, $\bar {v}(t)$, and an estimate of the magnitude of the Fourier transformed MUAP, $|\bar {H}(f)|$, which is basically the square root of the EMG power spectrum. The latter was estimated by means of the Matlab function PWELCH. Figure 7 illustrates that the algorithm uses the two types of data in a quite different way: $|\bar {H}(f)|$ is multiplied by |*R*(*f*)| to predict the magnitude of the Fourier transformed VEMP, whereas $\bar {v}(t)$ directly enters the calculation of a figure of merit (after transformation into the frequency domain).The figure of merit quantifies the difference between predicted and measured VEMP using the cost function defined in Eq. (16). This measure eventually determines the next action taken by the nonlinear optimization routine (i.e., either continuation with a new set of parameters or exit).

Constraints were implemented by means of parameter transformations. More specifically, to ensure that a certain parameter *a* is always greater than a lower limit *a*_0_, the optimization procedure internally worked with a parameter *α*, assuming that *a*=*a*_0_+*α*^2^. This way the model parameters in Eq. (18) were constrained as follows: *r*_*k*_≥−1 and *τ*_*k*_≥1 ms (1≤*k*≤*K*), and *t*_*k*_≥*t*_*k*−1_ (2≤*k*≤*K*).
